# Estimating the Frequency of Single Point Driver Mutations across Common Solid Tumours

**DOI:** 10.1038/s41598-019-48765-2

**Published:** 2019-09-17

**Authors:** Madeleine Darbyshire, Zachary du Toit, Mark F. Rogers, Tom R. Gaunt, Colin Campbell

**Affiliations:** 1 Intelligent Systems Laboratory, University of Bristol, Bristol, BS8 1UB United Kingdom; 2 Bristol Medical School, University of Bristol, Bristol, BS8 1UD United Kingdom; 3 MRC Integrative Epidemiology Unit (IEU), University of Bristol, Bristol, BS8 2BN United Kingdom

**Keywords:** Genetics research, Oncology

## Abstract

For cancers, such as common solid tumours, variants in the genome give a selective growth advantage to certain cells. It has recently been argued that the mean count of coding single nucleotide variants acting as disease-drivers in common solid tumours is frequently small in size, but significantly variable by cancer type (hypermutation is excluded from this study). In this paper we investigate this proposal through the use of integrative machine-learning-based classifiers we have proposed recently for predicting the disease-driver status of single nucleotide variants (SNVs) in the human cancer genome. We find that predicted driver counts are compatible with this proposal, have similar variabilities by cancer type and, to a certain extent, the drivers are identifiable by these machine learning methods. We further discuss predicted driver counts stratified by stage of disease and driver counts in non-coding regions of the cancer genome, in addition to driver-genes.

## Introduction

For common solid tumours, a number of variants in the coding regions of the human cancer genome can act as disease-drivers. Approximately, 95% of these variants are single-base substitutions with 90.7% resulting in missense change, 7.6% resulting in nonsense alteration, and 1.7% as alterations in regions adjacent to start and stop codons^1^. However, though there may be many genomic positions where variants can act as disease-drivers, an individual clone would only have a small subset of these. There have been various attempts to estimate the mean number of driver mutations in common solid tumours. Assuming mutation rates are constant throughout life, classic studies based on the relationship between age and cancer incidence led Nordling^2^ and Armitage and Doll^3^ to suggest approximately six or seven sequential mutational events. Alternative arguments have similarly suggested a limited number of driver mutations^4^. For example, Tomasetti *et al*.^5^ argued for only a few sequential mutations in a study of lung and colorectal cancer. Using observations based on rates of non-synonymous and synonymous base substitutions, Martincorena *et al*.^6^ have recently argued that the number of coding substitutions that are driver mutations is quite small, on average about 4 per tumour, across common solid tumours. They argue that tumour types can vary significantly from this average, from less than 1 for thyroid and testicular tumours, to more than 10 in coding regions for endometrial and colorectal tumours^6^. An exception to this picture is the high mutation count which can stem from alterations to the proofreading domains of DNA polymerases POLE and POLD1, leading to hypermutation. The basis of their model is the normalized ratio of non-synonymous to synonymous mutations (dN/dS) with the assumption that the large majority of synonymous mutations are neutral as disease-drivers, and thus can act as a proxy for the underlying null distribution of neutral variation. Since it is based on dN/dS, the model is restricted to coding regions. As reported by the authors^6^ estimation from dN/dS can be subject to bias affecting estimation. For example, it is sensitive to mislabelling of germline as somatic or vice versa, leading to downward or upward estimation bias respectively. Also, dN/dS is not an approach which lends itself readily to test accuracy estimation on independent unseen data, which is a standard validation step for the machine learning based methods we now discuss. However, the method proposed by Martincorena *et al*.^6^ is a helpful comparator model in the discussion below.

## Materials and Methods

In recent years a number of methods have been developed for predicting the pathogenic impact of variants in both the coding and non-coding regions of the human genome. For example, in Shihab *et al*.^7^ and Rogers *et al*.^8^ we developed such predictors based on pathogenic disease-driver single nucleotide variants (SNVs) from the Human Gene Mutation Database (HGMD^9^) and assumed neutral variants from the 1,000 Genomes Project Consortium (1000 G^10^). A variety of similar predictors have been proposed^11–16^. Catalogues covering documented instances of disease-traits associated with single nucleotide variants, across *non-cancer* diseases, indicate that approximately 90% of these are located in non-coding regions of the human genome^17^ and our previous tools predict a large proportion of disease-driver variants in non-coding regions in consequence. In Rogers *et al*.^18^ we proposed *CScape*, a method for predicting the disease-driver status of single nucleotide variants in the coding and non-coding regions of the human cancer genome (the method is more fully described in Supplementary Section 1). The pathogenic (positive) examples were constructed from somatic single point mutations from the COSMIC database^19^ and the neutral (negative) examples were constructed using SNVs from the 1000 Genomes Project^10^. Henceforth we refer to these types of disease-driver positives as *SNV-drivers*.

*CScape* uses integrative binary classification methodology from machine learning (Supplementary Section 1). It was not trained on, and does not predict on, mitochondrial or allosomal (X and Y) chromosomes since these yield fewer examples of drivers and are functionally different from autosomes. The allosomes have evolutionary characteristics (e.g. mutation rates) distinct from autosomal chromosomes, potentially impacting any features influenced by mutation rates, such as the sequence conservation scores used (see Supplementary Section 1). In addition, the mutational burden on chromosome X could be different between males and females suggesting development of a specialist predictor sensitive to this difference. However, a contribution to driver counts from allosomes is to be expected and this would be an interesting further extension of the current study.

We eschew a comparison with other such classifiers in the discussion below because in Rogers *et al*.^18^ we established an improved performance of *CScape* against MutationAssessor (MAS)^13^, PolyPhen2^11^, SIFT^12^, FATHMM-MKL^7^, CADD^14^, DANN^15^ and FunSeq2^20^. During the training process *CScape* could use up to 30 feature groups which may be informative about disease-driver status, though will typically use only a subset of these which are found to be informative (these subsets are different for prediction in coding and non-coding regions). The classifier used some feature groups from the Variant Effect Predictor^21^ representing allele consequences and affect on amino acids. For positives (disease-drivers, drawn from COSMIC^19^), we used a filtering threshold based on the highest recurrence level for a SNV observed in the COSMIC data which shows little evidence of bias and provides enough training examples to build a classifier. With this criterion we selected a recurrence threshold of *r* = 5 in coding regions and *r* = 3 in non-coding regions^18^. The classifier was tested using leave-one-chromosome-out cross-validation (LOCO-CV) in addition to being tested on previously unseen data from four datasets covering single point mutations in coding regions of the cancer genome, and two datasets covering mutations in the non-coding regions of the cancer genome. Using balanced test sets, and LOCO-CV testing, the classifier could achieve a test accuracy of 72.3% in coding regions and 62.3% in non-coding regions with some higher test accuracies on independent datasets. A confidence measure was associated with the predicted class label (disease-driver or neutral), denoted as a *p*- *score*^18^. This score was determined by Platt scaling^22^ and is on a range 0 to 1 (0 is maximum confidence of negative and 1 of positive). As one would expect, the test accuracy on high confidence predictions is higher than these baseline test accuracies, 72.3% and 62.3%, based on a 0.5 threshold on the *p*-score. With high confidence prediction, for example, we achieve a balanced test accuracy of 91.7% in coding regions and 76.1% in non-coding regions at *p*-score thresholds of 0.89 (coding) and 0.70 (non-coding) respectively. The positive predictive values (PPV) and numbers of true positives predicted indicate balanced predictive accuracy rather than a misleading test accuracy based on accurate inference with one class only (e.g. the negatives).

*CScape* predictions are available as an online resource http://cscape.biocompute.org.uk. A predictions database, software for querying the database and training and test datasets are all available for download from this website.

## Results

### Motivation for the study

We applied the *CScape* predictor to a variety of common solid tumour cancers to determine the mean and median number of SNV-drivers in coding regions of the cancer genome. We found a reasonable alignment with Martincorena *et al*.^6^ using test data derived from the International Cancer Genome consortium^23^ and labelled as primary tumours. To compare the means and medians of different types of cancer we will initially use samples which are not differentiated by stage of disease. However, we discuss the influence of stage of disease later in this Section, in the Discussion and the Supplementary.

With this assumption, and comparing means for cancer types in common between both analyses, we find that our lowest and third lowest ranked cancers by SNV-driver count are thyroid and renal cancer and these are lowest ranked by Martincorena *et al*.^6^. Martincorena *et al*.^6^ give bladder urothelial, uterine corpus endometrial and esophageal cancers as having the highest counts, for the means, and these are highest, third highest, and fifth highest for *CScape*, across the cancer types they consider. Breast cancer is an example of a cancer with a SNV-driver count towards the lower end of the range, with a mean of 6 coding drivers and Martincorena *et al*.^6^ similarly state a low mean of 4 coding driver mutations for this disease. Illustrative plots for thyroid and breast cancer are given in Supplementary Fig. 1, and a comparison across a variety of cancer types in Supplementary Fig. 2, all at the same value of the *p*-score threshold (0.9). *However*, we note that the choice of the *p*-score threshold influences the stated counts (cf. Supplementary Fig. 1): with a lower threshold on the confidence we let through more prospective positives and increase the count. This approximate agreement on the limited numbers of drivers involved, and the relative ranking of common solid tumours, is achieved despite the methodologies being quite distinct: *CScape* uses training data from two different sources, cancer data from COSMIC^19^ and data from the 1000 Genomes Project^10^, derived from healthy subjects. The test data used in the study below was derived from the International Cancer Genome consortium (ICGC^23^, cf. Supplementary Section 1). In contrast, Martincorena *et al*.^6^ use data derived from 7,664 tumours from The Cancer Genome Atlas^24^ (TCGA). *CScape* is based on an integrative binary classifier using a wide range of feature groups, while Martincorena *et al*.^6^ propose a statistical argument based on the dN/dS ratio. A small number of features used by *CScape* use data from the Variant Effect Predictor (VEP^21^) and are thus influenced by prospective amino acid substitution. The advantages of using an integrative machine learning classifier over an argument based on dN/dS, is that the classifier is able to predict which single point mutations are drivers, with an associated confidence score, the method implicitly uses a wide variety of data sources for this prediction and the method can be adapted to make predictions of the driver status of single point mutations in non-coding regions. In this study we removed samples with apparent hypermutation (more than 500 drivers), and a description of the data and method is presented in Supplementary Section 1.

### Estimations using a false discovery rate of 5%

**Figure 1:**
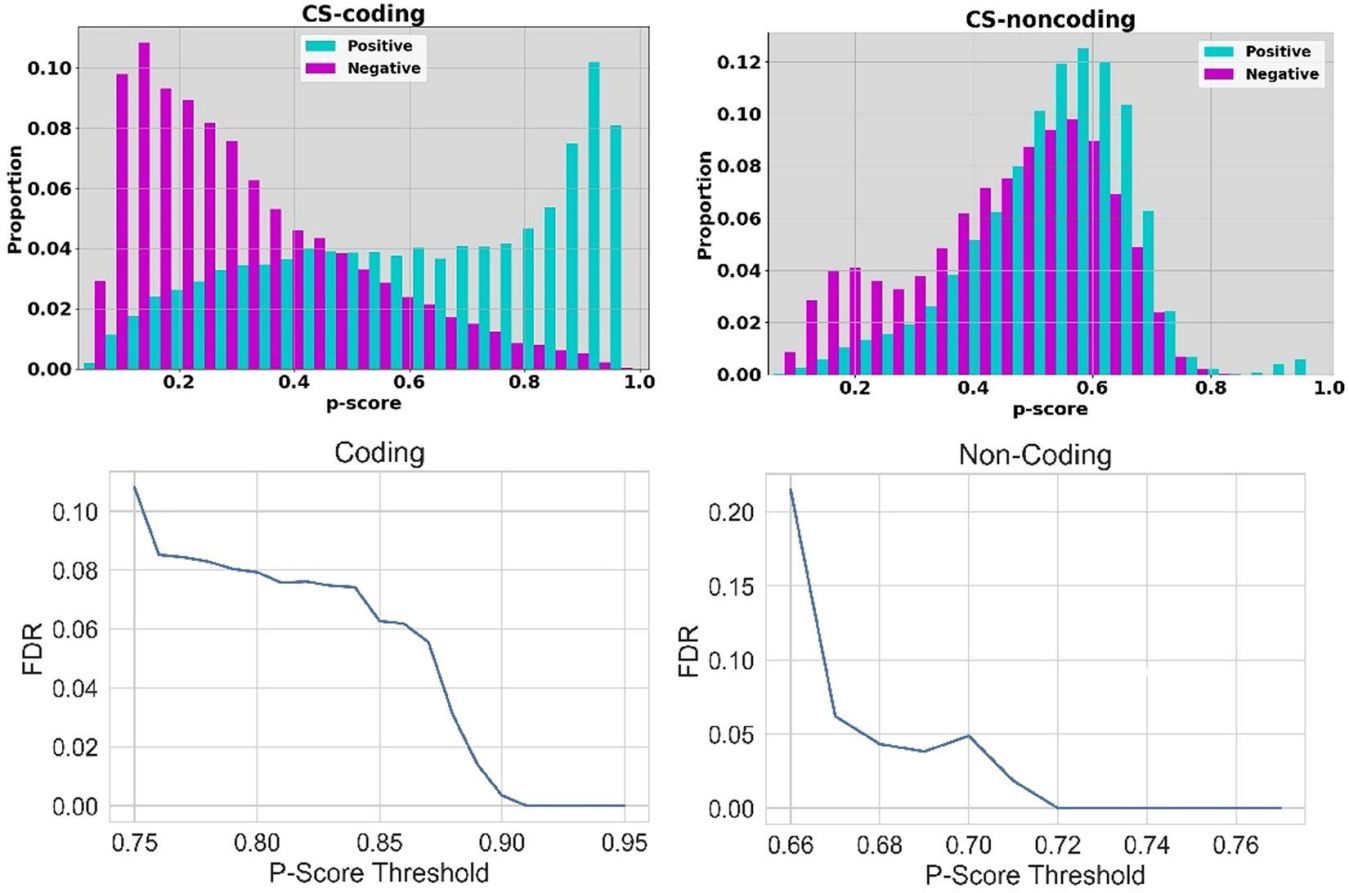
**Top, left**: the proportions (*y*-axis) for prediction of positives (disease-drivers) and negatives (neutral) at different values of the *p*-score threshold (*x*-axis), for predictions in *coding* regions of the cancer genome. **Top, right**: the proportions (*y*-axis) for prediction of positives (disease-drivers) and negatives (neutral) at different values for the *p*-score threshold (*x*-axis), for predictions in *non-coding* regions of the cancer genome. **Bottom, left**: The False Discovery rate (FDR, *y*-axis) versus the threshold on the *p*-score (*x*-axis) for prediction in coding regions. **Bottom, right**: The False Discovery rate (FDR, *y*-axis) versus the threshold on the *p*-score (*x*-axis) for prediction in non-coding regions.

**Figure 2:**
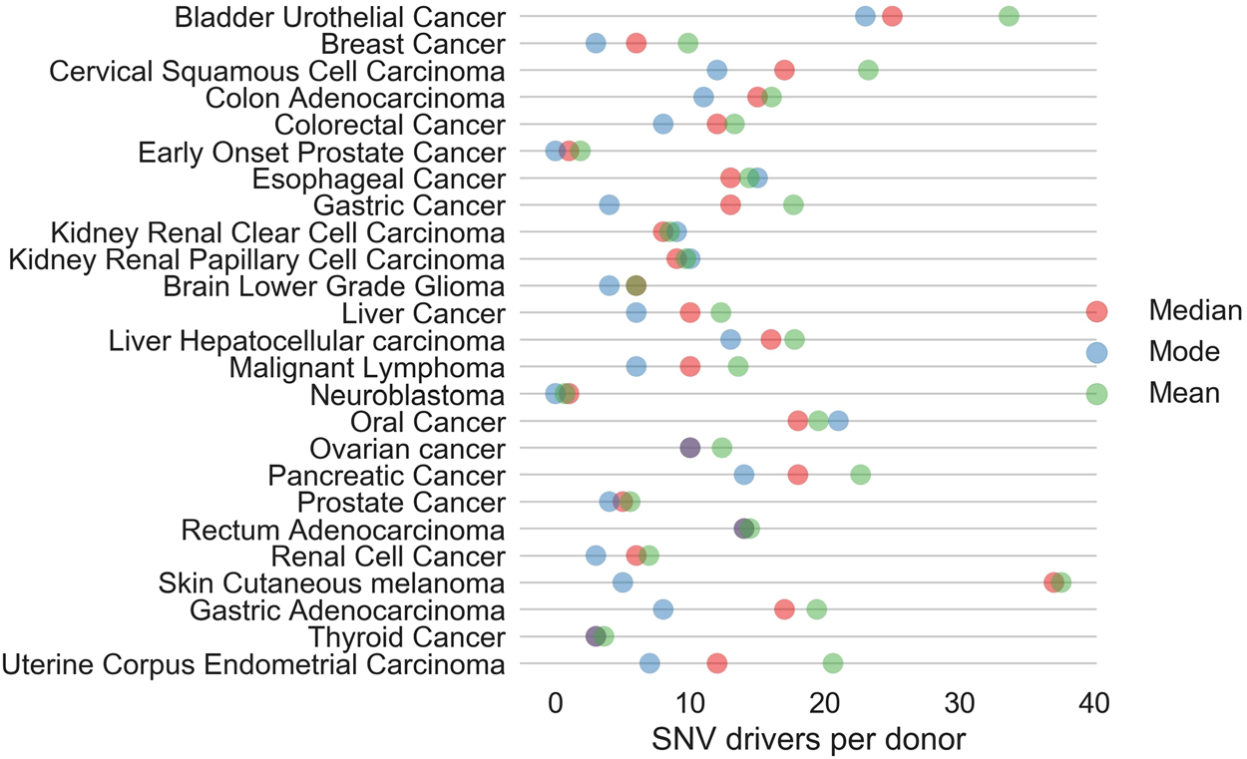
The estimated modes (blue), the medians (red) and the means (green) for coding SNV-drivers across a variety of common solid tumours. A few circles are other colours due to a co-incidence of these statistical measures. These results were generated using a false discovery rate of 5%, leading to a threshold of 0.88 on the *p*-score.

Given this motivating observation, in this paper we investigate disease-driver mutation counts, for single point mutations, across the genomes of a number of types of common solid tumour. We have noted that these mutation counts will be dependent on the selected threshold on the *p*-score. Determination of a significant threshold on a range of *p*-values is a well studied issue in many biomedical contexts. For example, with the analysis of expression array data, a spectrum of *p*-values is often obtained in an experimental context and the size of the set of differentially expressed genes is very dependent on the *p*-value threshold. Following discussion of the most suitable approach to threshold determination in the statistics literature^25,26^, our first approach would be to use the false discovery rate (FDR), as our criterion. In Fig. 1 (top row) we give the proportion of correct positive and negative predictions on test data for the two sub-classifiers within *CScape* (*CS-coding* and *CS-noncoding* for prediction in coding and non-coding regions respectively, see Rogers *et al*.^18^). Whereas *CS-coding* could be viewed as reasonably successful overall and apparently accurate with high confidence prediction, *CS-noncoding* is weak overall because of our current lack of understanding of the pathogenic impact of variants within non-coding regions of the cancer genome. From a statistical viewpoint and using balanced test data, we can use integrated proportions beyond a given threshold value from these histograms as a proxy for true and false predictions, and hence we can estimate a FDR for a given threshold on the *p*-score (Fig. 1). With this first attempt we will use a conservative FDR of 5%. This criterion gives threshold values of 0.88 (coding) and 0.68 (non-coding). Given this choice for the FDR, in Fig. 2 we give the modes, mean and median numbers of SNV-drivers in coding regions of the genome, across a number of types of cancers. Thyroid cancer has a very low median for the SNV-driver count and the cancer with the highest median is bladder urothelial cancer. Some of these results are consistent with our understanding of distinct cancer types. For example, samples associated with skin cutaneous melanoma exhibit a large difference between the mode and median suggesting an extensive heterogeneity which could arise from an abundance of single point mutations induced by ultraviolet (UV) sunlight exposure.

Mutations driving cancer are typically random in occurrence and the mode of the distribution could be expected to lie at a lower SNV count than the median, as would be the case for a Poissonian distribution (excepting hypermutation, relatively fewer numbers of examples are observed with relatively larger mutation counts). Given the existence of subtypes of various categories of common solid tumour, this distribution of counts is likely multi-modal in general. In Fig. 2 we plot the primary mode (the highest peak in a histogram) and the median across a number of common solid tumour types. As expected, the mode is generally lower than the median. This observation suggests that there are potentially significant numbers of patient tumours with smaller driver sets than suggested by the medians we have presented (we call these *neo-modal* populations).

### Estimations independent of the *p*-score threshold

**Figure 3:**
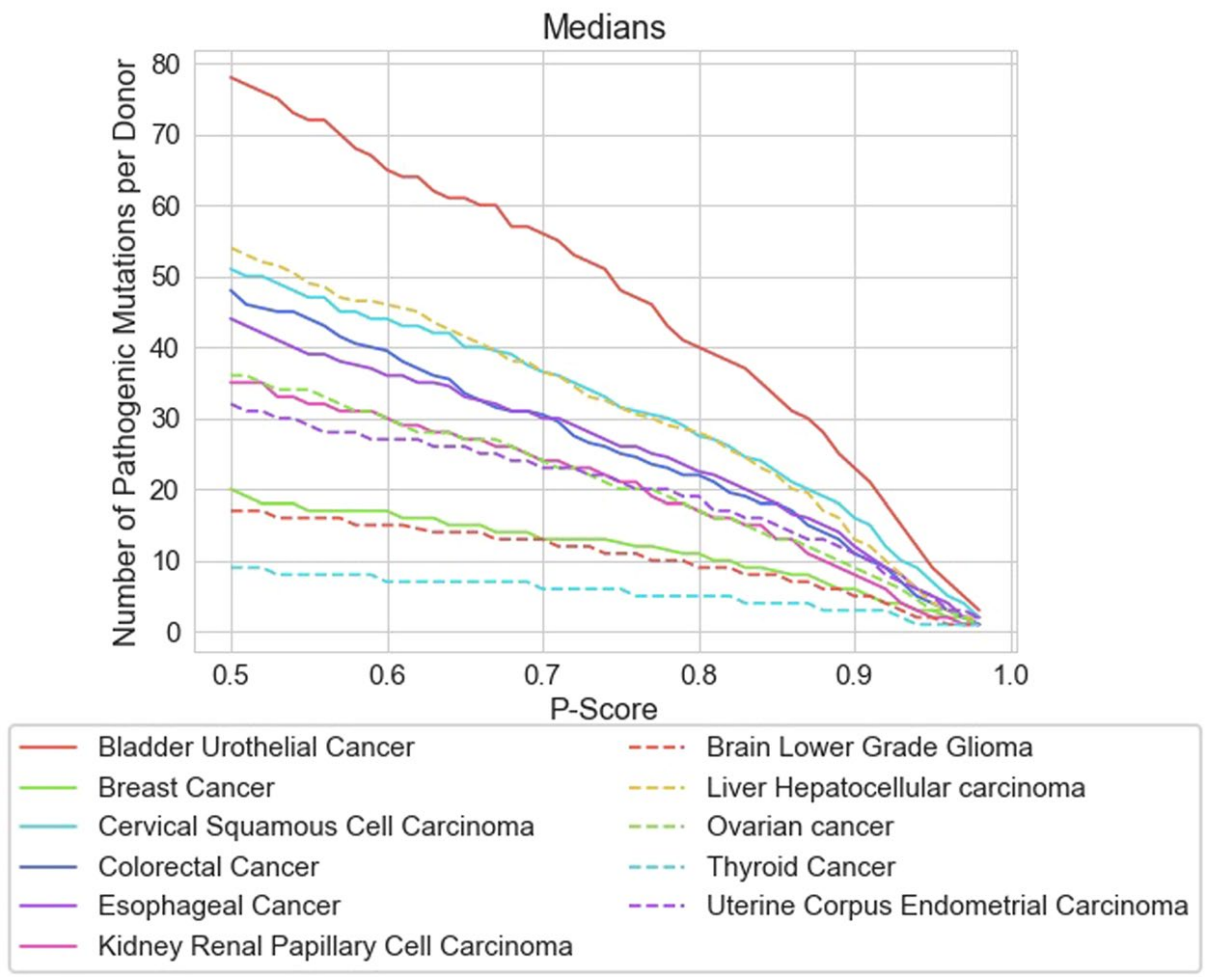
Plots of the medians across a range of common solid tumours for the counts of disease-driver SNVs in coding regions of the cancer genome. The *y*-axis gives the median count of disease-driver single point mutations, the *x*-axis gives the *p*-score threshold. Plots covering a wider variety of common solid tumours or using the mean counts instead are presented in Supplementary Section 3.

We can also gain useful insights which are *independent* of our criterion for choosing a FDR or *p*-score threshold. *Firstly*, in Fig. 3, we give the medians for a selection of common solid tumours plotted across a range of *p*-score threshold values. Further plots are presented in Supplementary Section 3 covering a broader range of cancers, in addition to considering the mean counts. The given curves do not typically cross each other so the relative ranking of different types of cancer, according to SNV-driver count, is preserved and largely independent of the *p*-score threshold used. We consistently find that thyroid cancer has a very low SNV-driver median count and bladder urothelial cancer has the highest. We may use the non-parametric two-sided Mann-Whitney *U* test to determine the *p*-value for the hypothesis that the SNV-driver count sets are representative of the distribution across all types of cancer rather than forming a distinct population for the given cancer type. The sample sizes per cancer type are generally large in number (Supplementary Table 1, 528 samples for thyroid cancer, for example) and differences between medians and means quite large. Thus, as expected, these *p*-values are statistically significant. Taken against the reference of the whole population across all cancers, but excluding the given cancer type, the *p*-value for thyroid cancer is upper bounded by 10^−100^, for example, for a calculation based on a FDR of 5%. Thus this differentiation for SNV-driver counts between different types of cancer is very significant.

**Figure 4:**
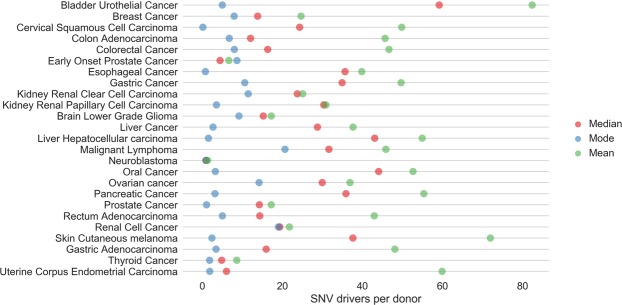
The estimated modes (blue), the medians (red) and the means (green) for coding SNV-drivers across a variety of common solid tumours. These results do not depend on a choice of the FDR or *p*-score threshold. In this estimation we have used histogram bins of size 0.01 for the *p*-scores with normalisation of counts within each such bin to provide estimates of *p*(*n*_*i*_ = +1|*c*_*i*_ = +1) and *p*(*n*_*i*_ = −1|*c*_*i*_ = +1).

*Secondly*, we can also acquire a basic estimate of the SNV-driver count which is independent of a choice of FDR or threshold on the *p*-score. For a variant at position *i* and a given mutation (e.g. *A* → *C*), let *c*_*i*_ = +1 denote classifier predicts a driver, *n*_*i*_ = +1 denotes *it is* a driver. Similarly, *c*_*i*_ = −1 denotes neutral prediction, and *n*_*i*_ = −1 denotes the variant *is* neutral. As before, for prediction in coding regions, we can use the relative proportions in a bin (cf. Fig. 1) to estimate *p*(*n*_*i*_ = +1|*c*_*i*_ = +1) and *p*(*n*_*i*_ = −1|*c*_*i*_ = +1), thus using the test data as a proxy for believing *n*_*i*_ = +1 or *n*_*i*_ = −1 and noting that these prediction counts have been derived from 50:50 balanced test data. We include a contribution to the count when *p*(*n*_*i*_ = +1|*c*_*i*_ = +1) > *p*(*n*_*i*_ = −1|*c*_*i*_ = +1) i.e. for a given mutation at position *i*, the classifier would more likely correctly predict a variant would a driver, rather than neutral. Assuming this eligibility for inclusion in the count, if a *p*-score falls within a given bin in the histogram then its contribution to the count is (+1)*p*(*n*_*i*_ = +1|*c*_*i*_ = +1) + (−1)*p*(*n*_*i*_ = −1|*c*_*i*_ = +1) and we integrate these weighted predictions. This allows for the inclusion of less confident estimations of disease-driver status, though weighted by the factor that these predictions may, indeed, be false positives. We sum up the counts this way and the corresponding modes, means and medians across a variety of common solid tumours are given in Fig. 4. As expected, the means and medians for the SNV-driver counts increase over the more conservative results in Fig. 2. Due to the different distribution and estimation of the primary mode relative to Fig. 2, the modes are still frequently low. However, even with this less conservative estimation method, the number of SNV-drivers remains low and in the region of double digits at most.

### The influence of stage of disease

So far, in order to compare different types of cancer, we have amalgamated samples for a given type, independently of stage of disease. In Supplementary Section 4 we tabulate the means and quartiles for SNV-driver frequency counts predicted by *CScape*, where the cancer has been stratified by stage of disease. The prospective accumulation of SNV-driver mutations with stage of disease has been argued by many authors, though frequently via observation of *tumour mutational burden* (TMB), the number of non-synonymous mutations in coding regions of the cancer genome. We can also define an estimated *tumour driver burden* (TDB), as the number of estimated drivers in coding regions. Since many non-synonymous mutations are not disease-drivers, the TDB is obviously more insightful, but requires a method to separate passengers and drivers. The paper of Martincorena *et al*.^6^ attempts such a distinction and they include a study (p. 1036 of ^6^ and their Fig. S4C) to evaluate whether the number of driver mutations significantly increases as cancers progress. This is achieved via driver estimates for stage I and stage IV tumours. Interestingly, they find no significant differences in the number of estimated drivers. Our results presented in Supplementary Section 4 are in general agreement with this observation. Thyroid (typecode: THCA) and renal cell (RECA) cancers have low TDBs which remain approximately constant with stage. Similarly, esophagal (ESCA) and gastric cancer (GACA) do not appear to accumulate drivers with stage of disease. Cancers in our analysis, not covered in^6^, and also with no evidence of accumulation of drivers with stage, are malignant lymphoma (MALY), pancreatic cancer (PACA) and neuroblastoma (NBL). The latter has a very low TDB which remains static according to stage. Possible exceptions to this picture are colorectal (COCA) and liver cancer (LICA) which may demonstrate increasing TDB with stage, according to our analysis. We observe a consistent monotonic accumulation of SNV-drivers with stage of disease for prostate cancer (both EOPC and PRAD). Hence, accumulation of SNV-drivers with stage of disease appears as an exception, across different types of cancer, rather than a general trend.

### Genes acting as disease-drivers

An interesting further question is the identification of those genes where these SNV-drivers are commonly located. As a machine learning based method, a requisite starting point will be demonstrated validation of the method on unseen test data. Hence we start by illustrating classifier performance on the top 50 identified prospective SNV-drivers reported in the comprehensive study of Rheinbay *et al*.^27^, where we select coding drivers, non-coding drivers will be considered later. This study used data from the ICGC and TCGA Pan-Cancer Analysis of Whole Genomes (PCAWG) consortium, giving a dataset of more than 2,700 cancer genomes derived from more than 2,500 patients. Since *CScape* was trained on cancer-specific COSMIC data^19^, and 1000 Genomes^10^ for the healthy controls, the PCAWG dataset acts as an independent evaluation. The confidence measures for *CScape* prediction are presented in Table 1 with a restriction to coding SNV-drivers in the autosomes of cancer genomes. One of the most common SNV-drivers is in the gene *KRAS* which expresses the K-Ras protein. This protein functions in the RAS/MAPK pathway which is hyper-activated in up to 30% of all cancers^28^. *NRAS* is another member of the RAS subfamily, inclusive of *HRAS* and *KRAS*, and has been implicated in many human cancers^29^. The involvement of the *PIK3CA* gene in a wide variety of common human cancers has been well supported by many studies^30^. Somatic mutations in the well-known tumour suppressor gene *TP53* are one of the most frequent alterations in human cancers^31^. Finally *IDH1* is an example of a gene with more specific effects in certain types of cancer. Thus somatically acquired mutations have been implicated in gliomas and chondrosarcomas^32^. Excepting *TP53*, *CScape* predicts that all these single point mutations are disease drivers with confidence levels equal to, or exceeding, 0.829. *TP53* is different in that any alteration at some locations will act as a disease-driver (e.g. 17:7577538). However, at other locations, the type of alteration involved is important. Thus, at 17:7577094, *G* → *A* is a predicted oncogenic driver and *G* → *T* is predicted benign.

**Table 1:** The top recurring single point driver mutations in coding regions proposed by Rheinbay *et al*. (Extended Data Fig. 1 in^27^).

Gene	Chr.	Position	Ref.	Mut.	Confidences
*KRAS*	12	25398284	C	{A, G, T}	{0.922, 0.916, 0.910}
*KRAS*	12	25398285	C	{A, G, T}	{0.931, 0.915, 0.902}
*PIK3CA*	3	178952085	A	{C, G, T}	{0.854, 0.804, 0.829}
*PIK3CA*	3	178936091	G	{A, C, T}	{0.959, 0.949, 0.991}
*TP53*	17	7577120	C	{A, G, T}	{0.887, 0.920, 0.876}
*TP53*	17	7577538	C	{A, G, T}	{0.932, 0.951, 0.909}
*TP53*	17	7577094	G	{A, C, T}	{0.819, 0.853, 0.322}
*TP53*	17	7578212	G	{A, C, T}	{0.989, 0.854, 0.443}
*TP53*	17	7578190	T	{A, C, G}	{0.926, 0.916, 0.915}
*TP53*	17	7577539	G	{A, C, T}	{0.877, 0.907, 0.459}
*TP53*	17	7577121	G	{A, C, T}	{0.760, 0.875, 0.717}
*NRAS*	1	115256530	G	{A, C, T}	{0.987, 0.911, 0.931}
*IDH1*	2	209113112	C	{A, G, T}	{0.940, 0.957, 0.926}

Their study uses data from the Pan-Cancer Analysis of Whole Genomes Consortium and uses in excess of 2,700 cancer genomes from more than 2,500 patients. This table only gives single nucleotide variants located on autosomes and labelled by our classifier as residing in coding regions (the corresponding table for non-coding mutations is presented in Supplementary Section 6). The table presents the gene, chromosome (Chr.), position and reference nucleotide (Ref.) based on the GRCh37 reference genome. The three prospective variants are presented (Mut.) with the confidence driver-status given in the next column, in the same relative order, and derived from our predictor *CScape* (http://cscape.biocompute.org.uk).

In terms of the broader set of cancer genes implicated by *CScape*, we note that the classifier was trained on assumed SNV-drivers derived from the COSMIC database. Observed recurrent somatic point mutations restricted to cancer samples will act as positives in the training data and will therefore largely reside in genes already documented in the COSMIC Cancer Gene Census^33^. As a machine-learning based tool, however, *CScape* will be able to *generalize* and make predictions to unseen examples not in its training set. An example would be the gene *PRKCA*. A recurrent point mutation at chromosome 17, position 64738741 *G* → *C*, introduces a D463H amino acid substitution and this has been described as a hallmark of chordoid glioma^34^. The authors of the relevant study^34^ comment that a query of the COSMIC and *cBioPortal* databases found no PRKCAD463H mutation referenced and this gene is not currently (2019) in the COSMIC Cancer Gene Census. However, *CScape* (GRCh37 reference) predicts this point mutation as oncogenic with high confidence (0.964).

Using *CScape* we can also choose a particular cancer and rank genes according to the relative frequency of occurrence of SNV-drivers. As an example, suppose we select *pancreatic cancer* and, using ICGC data, we rank genes according to the percentage of samples which have at least one SNV-driver predicted with a confidence (*p*-score) equal to or exceeding 0.88 (hence associated with a false discovery rate of 5% or less). We find the top ranked gene is *KRAS* (incidence of 86.5%), followed by *TP53* (41.0%), *SMAD4* (12.9%), *TTN* (6.3%) and *TNN-AS1* (5.8%). The role of *KRAS* in pancreatic cancer is well documented^35^. The requirement of a minimum of *one* such high confidence SNV-driver may appear low, but we have been arguing that there are a very small number of such SNV-drivers in a typical cancer genome. Hence a frequency count taken across multiple samples, with this criterion, will highlight significant driver-genes by type of cancer.

A listing of the top five genes containing at least one of these high confidence SNV-drivers is presented in Table 4 to 8 of the Supplementary, across 25 types of cancer (the full listing is at^36^). Most of the listed genes are well known in their respective contexts. Though the percentages differ by type of cancer, *TP53* manages to qualify in the top five genes for 17 of 25 types of cancer. Other genes which feature across multiple types of cancer and enter the top five driver-gene list are *PIK3CA* (in 6), *KRAS* (in 5) and *CTC-297N7.11* (in 4). *APC*^37^ appears as the top ranked driver gene for colon adenocarcinoma (COAD) and colorectal (COCA) cancer and the genes and comparative ranking of all top five genes is the same between these two (despite usage of different tumour samples). *BRAF* also qualifies in the top five cancer genes in two instances: skin cutaneous melanoma and thyroid cancer. Mutations in *BRAF* are well known and studied in the context of melanoma^38^. Thyroid cancer has these types of SNV-drivers present in *BRAF* in 55.8% of cases, the next qualifying genes being *TTN*, *TTN-AS1*, *NRAS* and *KMT2C* all with a low incidence rate of 1.3%. The relevance of *BRAF* in thyroid cancer has been noted by a large number of authors e.g.^39^.

Several genes are highly mutated but are usually excluded from candidate cancer driver lists^40^. These genes are mostly extremely large and they include *Titin* (*TTN*), the gel forming mucins (*MUC*) and ryanodine receptors (*RYR*)^41^. Apart from size, which increases the probability of accumulating false positive predictions, argument is weighted against giving *TTN* driver status since it is commonly mutated in non-cancer tissue^42^ and expression is restricted to muscle protein formation, among other issues. However, *TTN* is a complex gene and it co-occurs in our lists with *TTN* antisense RNA 1 (*TTN-AS1*), the latter pairing with *TTN*, at an equal, or slightly reduced frequency of occurrence. *TTN-AS1* is a long non-coding RNA gene which is transcribed from the opposite strand to the *Titin* gene. *TTN* mutations have been reported as highly predictive of patient survival with some cancers^41^ and *TTN-AS1* has been recently argued as an oncogene across several cancers^43–46^.

The above mentioned driver-genes are generally well studied in cancer research and are typically highly ranked using methods based on a count of observed non-synonymous single point mutations across cancer genomes. However, the percentage occurrence of mutations in genes tends to tail rapidly and it is in this tail of low incidence rates for given SNVs where *CScape* can be most useful in distinguishing passengers from drivers. On the Help/Documentation webpage of the *CScape* website^36^ we present a longer list of ranked genes (file: *driver-genes*) detailing these tails of driver-genes across the 25 types of cancer considered here, using the same FDR of 5%. In Rogers *et al*.^18^ we also investigate contiguous nucleotide sequences of high confidence predictions for driver-status (called *Regions of Interest*). This study highlighted 191 autosomal genes, much less studied in the cancer literature and not typically marked by common mutations.

**Figure 5:**
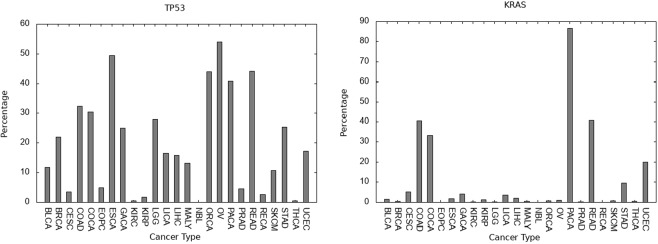
The percentage incidence (*y*-axis) for the selected gene having at least one SNV-driver, determined with a false discovery rate of 5% for **Left**: *TP53*, **Right**: *KRAS*. Data is from the International Cancer Genome Consortium and the *x*-axis gives the typecode for the given type of cancer (see Supplementary Table 1).

Apart from selecting a cancer type and ranking genes within the type, the other way of portraying data is to select a gene and estimate its influence as a driver-gene across different cancer types. In Fig. 5 we portray the percentage incidence for having at least one SNV-driver, at a false discovery rate of 5%, for the genes *TP53* and *KRAS* across 25 cancers, as an illustration.

### Drivers in non-coding regions of the cancer genome

As commented in the beginning, for *non-cancer* disease, the ratio of documented SNV-drivers in non-coding regions over coding regions, suggests the large majority of SNV-drivers are located in non-coding regions. Some studies have argued that cancer genomes do not follow this trend and that the ratio of drivers in non-coding regions to coding is relatively smaller^27^. We do not regard *CScape* predictions in non-coding regions of the cancer genome as reliable enough to suggest a resolution of this issue (Fig. 1, top right subfigure). However, there are a number of single-point mutations in non-coding regions which have been verified as disease-drivers and useful insight will be gained from considering these. As we have previously discussed^18^, three mutations in the *TERT* promoter region have been characterised as disruptive to putative ETS (E26 transformation specific) family transcription factor binding sites^47–49^ and are verified drivers. Five additional recurrent driver mutations in regulatory elements upstream of *SDHD* and *PLEKHS1* have been documented^49^. *CScape* correctly predicts 7 from 8 of these mutations as putative drivers^18^ with confidence measures in the range 0.52 to 0.81. The exception is one mutation for *SDHD* which is outside the core response element. This performance is only matched by *DANN* for the methods used in our comparison^18^.

We have extended this analysis to the larger set of non-coding SNV-drivers proposed in the Pan-Cancer Analysis of Whole Genomes (PCAWG) study of Rheinbay *et al*. (cf. Extended Data Fig. 1 in^27^). We use their top 50 list of proposed coding and non-coding SNV-drivers, restricting to autosomes and locations which our predictor labels as *non-coding*. The results are tabulated in Supplementary Table 9. Of the 31 non-coding locations considered, 23 point mutations are predicted *oncogenic* by *CScape*. At 7 further locations the prediction is labelled *possibly oncogenic*, that is, the predicted driver status label is oncogenic or benign depending on the type of mutation which has occurred. Only in one instance is the predicted label *benign* (1:10359944). This is a good level of prediction performance, noting that *CScape* was originally trained and tested on approximately balanced datasets in terms of class label^18^, and therefore does not have an implicit bias towards positive, disease-driver, prediction. However, for those point mutations where the prediction is oncogenic, covering the three possible alterations, 64 lie in the confidence band of 0.5 → 0.7 and 19 have a confidence greater than 0.7. The implication is that the large majority of non-coding oncogenic predictions lie in regions of weak confidence for the classifier (Fig. 1, top right subfigure). This allows for substantial numbers of false positives and false negatives and therefore a high expected variance in the frequency count of SNV-drivers in non-coding regions. Any restriction to using a reasonable confidence level for oncogenic status could lead to misleading under-estimation. Applied to cancer genomes, *CScape* often makes other positive predictions in the confidence band 0.5 → 0.7 beyond the sites given in Supplementary Table 9, and this leaves open the prospect of further non-coding drivers. Thus, an accurate estimate of the frequency count of SNV-drivers in non-coding regions awaits the development of more accurate predictors in non-coding regions, possibly specific to the functional structures lying in these regions.

## Discussion

Though SNV-driver sets appear small in size, various factors could lead to *under-estimation* of the driver count or create problems with interpretation. The *CScape* classifier was trained on assumed positives observed to have a high recurrence among a large number of tumours (*r* ≥ 5 in coding regions and *r* ≥ 3 in non-coding regions) while being absent from the neutral control samples. However, a tumour suppressor gene, for example, could be inactivated by SNVs scattered at very low recurrence rates per nucleotide position across a genomic region, such as an exon. Though functionally active in disease, the low frequency of occurrence of such variants means that they would be labelled negative (neutral) in both training and test sets. This shortfall in estimation can only be addressed with larger datasets and improved methodology for more accurately determining, potentially region-variable, recurrence levels for the labelling of a variant as a disease-driver.

There is also a potential bias leading to *over-estimation* of the SNV-driver count. In order to avoid bias, the *CScape* classifier was trained on approximately balanced data, in terms of positive disease-drivers and negative neutrals. This means that the number of false positive and false negative predictions should be approximately balanced. Evidently, the number of positive disease-driver SNVs is very small and this could lead to a bias towards *over-estimation*. We can understand this type of bias with an invented example. Thus, suppose we have an illustrative dataset with only 0.001% positives, the rest labelled negative. A predictor with very limited test accuracy, but balanced for false positives and false negatives, would predict approximately half the test datapoints as positives, even if these are with very low confidence. This bias explains the rising median estimates with lower *p*-score thresholds, portrayed in Fig. 3, for example. With a lower threshold on the *p*-score, we allow for more errors and therefore more false positives. Unfortunately, this type of bias can only be fully removed by the development of a classifier which is totally accurate on all test data.

Bias created by dependence of SNV-driver set sizes on the *p*-score cutoffs has also been an issue we discussed. In the Results Section we partly answer this issue with presentation of results independent of a threshold choice. We can also restrict to a false discovery rate of 5%. Relative to some other approaches, for example, Martincorena *et al*.^6^, our method has advantages of direct estimation by identification of SNV-drivers, together with independent validation of the methodology on unseen test data. However, this issue is a drawback and raises the consideration of possible further models which may fix a threshold with reduced ambiguity, while also prospectively identifying those variants driving disease. Such strategies would be possible, if an alternative criterion becomes available which is viewed as more accurate, but gives only genome-wide or gene-level estimations of driver counts. For example, suppose we wish to conform with the dN/dS argument of Martincorena *et al*.^6^. In this case one would minimise an objective function consisting of the sum of the absolute differences (or squared errors) between the predicted driver counts per gene and their number of driver mutations predicted per gene derived from dN/dS. This can be achieved by iteratively pursuing a one-dimensional line search for the optimal *p*-score cutoff threshold. This threshold could be cancer-type specific but would provide predictions of actual driver mutations in concordance with such an alternative model.

Our analysis of SNV-driver frequency counts by stage of disease also covered a different possible source of bias. Cancers can be initially detected at different stages of the disease, on the average, and successful intervention may reduce the population of late stage disease, which is therefore not fully represented. This leads to different sample sizes for different stages of disease (Supplementary Section 4) which in turn creates a possible bias in the use of a stage-independent mean frequency count comparison across different types of cancer. Our study indicated that for some types of cancer there is further accumulation of SNV-drivers as the disease progresses. However, for the large majority of cancer types, our study was in general alignment with Martincorena *et al*.^6^, in suggesting very limited accumulation of further SNV-drivers as the disease progresses through stages. For these cancer types, stage-independent estimation is therefore sound. Cancers where stage has an influence on driver count may be liver cancer and colorectal cancer with apparent rising SNV-driver counts by stage. For colorectal cancer this may conform with multi-step models for this disease^1^. Allowing for this rising trend, bladder urothelial cancer may not be particularly exceptional for a high driver count (Supplementary Section 4). For example, Figs 2 and 4 suggest prostate cancer (EOPC and PRAD) has low SNV-driver set sizes. However, for late onset disease (PRAD), there is a suggestion in the data (Supplementary Section 4) that stage IV prostate cancer may have larger coding SNV-driver sets sizes than bladder urothelial cancer (BLCA): it is just that the acquisition of prostate cancer data (PRAD) has been dominated by stage II tumour samples.

## Conclusion

Despite these various possible biases affecting driver count estimation, a number of insights appear sound. *Firstly*, some types of cancer have exceptionally low mean counts for the numbers of coding SNV-drivers, at levels which are very statistically significant. This observation can be applied to thyroid cancer, neuroblastoma and renal cancer, and there appears little further accumulation of SNV-drivers as these cancers progress to later stages of disease. Thyroid cancer was particularly remarkable with its low driver count and substantial influence of the gene *BRAF* as a driver. As expected, there are very statistically significant differences between driver counts for individual genes, for a given type of cancer, and in the prominence of a gene across different types of cancer.

*Secondly*, the above analysis supports the general message proposed by other groups^4,6,27^, that the mean number of coding SNV-drivers for most cancers is typically very small in size, either single digits or low double digits. Even for those cancer types where the mean size of coding SNV-driver sets is larger (double digits), there exist significant populations of tumours where, again, the coding SNV-driver set size is small and commonly back in the region of single digits (the neo-modal signatures discussed earlier). This observation is accentuated by our preceding discussion of an over-estimation bias stemming from the use of a partially accurate balanced classifier for estimation where one class (here, the positives) is very small in relative size. Taking note of this latter observation, the actual SNV-driver set sizes could be very small indeed, prospectively single digits on the average (excluding hypermutation), a view argued by authors cited in the Introduction.

The *third* observation is that machine learning methods, e.g. *CScape*, can be partially successful in identifying members of these driver sets (cf. Table 1 and Supplementary Table 9). The indicated low driver set sizes, and the ability to partially identify members of these small sets, suggests an ability to identify many of the core variants in the cancer genome which drive the development of a particular tumour.

The *fourth* observation is that these are machine learning methods under discussion and, as such, they can *generalize*. In the Introduction we mentioned 72.3% (coding) and 62.3% (non-coding) balanced test accuracies for *CScape*. In this case we remove *any* training examples which may have entered the test set, and evaluate test accuracy on this filtered set. For zero training error, the re-presentation of a training example to the *CScape* classifier will lead to correct categorisation, resulting in a positive selection for correct prediction. Highly recurrent positive point mutations within the COSMIC database will also likely feature as highly recurrent point mutations in the PCAWG data discussed earlier and hence not all examples within the PCAWG dataset may effectively qualify as unseen to the classifier. Even if the PCAWG dataset is new to the classifier, this explains the strong reported performances in Table 1 and Supplementary Table 9. However, the classifier is also able to generalize to unseen instances with a non-trivial accuracy. This observation becomes important when we note that our study of cancer genes in the Results Section, highlighted a number of well known driver-genes (*TP53*, *KRAS*, *BRAF*, etc) together with long tails of infrequent drivers^36^ which are most likely unique to an individual tumour and patient. It is in these tails of previously unseen instances where the classifier would be able to correctly label driver-status with some degree of accuracy.

The above study can be extended in many directions. One obvious choice is to pursue a parallel study of indel frequency counts across different types of cancer. Thus, for neuroblastoma, for example, our analysis suggests that single point driver mutations barely play a role and other categories of driver must be relevant. Indels are more substantial alterations to the genome, relative to SNVs, and predictors proposed in this context, e.g.^50^, have accordingly higher test accuracy. Another direction highlighted by our discussion has been the necessity to create improved predictors for SNVs in non-coding regions of the cancer genome. Other areas for investigation would be the role of intra-tumour heterogeneity, investigating additional SNV-driver contributions from the allosomes and the differences between identified subtypes of cancer e.g. breast cancer has clinically relevant subtypes^51^ and sub-stratification of driver-counts would be expected.

## Supplementary information

Supplementary Information
