# Supplementary material for: Estimating the Frequency of Single Point Driver Mutations across Common Solid Tumours
Source: Sci Rep. 2019 Sep 17;9:13452. doi: 10.1038/s41598-019-48765-2 (PMC6748970; doi:10.1038/s41598-019-48765-2)
Supplement: Supplementary file 1 — Supplementary Information [file 41598_2019_48765_MOESM1_ESM.pdf]

Supplementary Material for 'Estimating the Frequency of Single Point Driver Mutations across Common Solid Tumours'

Madeleine Darbyshire [1], Zachary du Toit [2], Mark F. Rogers [1], Tom R. Gaunt [3] and Colin Campbell [1]

[1]Intelligent Systems Laboratory, University of Bristol, Bristol, BS8 1UB, United Kingdom
[2]Bristol Medical School, University of Bristol, Bristol, BS8 1UD, United Kingdom
[3]MRC Integrative Epidemiology Unit (IEU), University of Bristol, Bristol, BS8 2BN, United Kingdom

Correspondence should be addressed to C.C. (email: C.Campbell@bris.ac.uk)

1 Method and Datasets

The *CScape* classifier is described in Rogers *et al* [1] and is trained with positive (disease-driver) variant data from COSMIC [2] and neutral variant data from the 1000 Genomes project [3]. The classifier presents an associated confidence measure, or *p*-score, on the range 0 for neutral to 1 for disease-driver, thus 0.7 means an 70% probability of neutral. This predictor is available at *http://cscape.biocompute.org.uk/*. *CScape* has sub-classifiers covering prediction in coding and non-coding regions of the cancer genome (*CS-coding* and *CS-noncoding*). To construct this classifier we investigated a variety of kernel-based methods [4] using the *scikit*-learning package (version 0.17.1). We found that gradient boosting [8] gave the best performance on validation data. We used a variety of different data sources to train the classifier, which are more fully described in our earlier paper [1]. For example, for *coding* prediction we used feature groups labelled *VEP* (variant effect prediction, inclusive of amino acid substitution), *46-way* and *100-way* conservation, *genomic context* measures and *spectrum* kernels [4] with the latter covering genomic sequence information. Thus single nucleotide variants in genomic regions which are highly conserved across species are more likely to be functional in human disease, relative to variants in regions where there has been significant sequence variation across species. This observation is then used as a possibly informative data source via the feature groups *46-way conservation* and *100-way conservation* (the split between these two is based on the range of species considered). For *non-coding* prediction we used feature groups such as *46-way conservation*, *100-way conservation*, *spectrum*, *genomic context* and *mappability* (the latter measuring the uniqueness of a region), for example. To incorporate these feature groups and construct the sub-classifiers, *CS-coding* and *CS-noncoding*, we used a greedy sequential learning approach based on leave-one-chromosome-out cross-validation (LOCO-CV). Thus we can order different prospective data sources according to accuracy on unseen validation data. Via a greedy approach we start by combining the two top-ranked data sources into a single kernel [4] and record its balanced accuracy according to LOCO-CV. We then add further prospective data sources in descending order of balanced accuracy, constructing a kernel for each combination of data sources. We terminate this greedy sequential addition of data sources if the balanced validation accuracy reaches a plateau or starts to decline. This terminates the learning process and therefore we can proceed to evaluation on unseen test data.

To derive predicted SNV-driver counts we used unseen test data from the International Cancer Genome

Consortium [5]. In Supplementary Table 1 we present the total sample sizes, number of hypermutator samples excluded and the number of zero-counts (i.e. for the given threshold on the p-score no disease-driver single nucleotide variants were predicted). The hypermutator estimations and zero-count estimations in Table 1 correspond to a threshold on the p-score determined by a FDR (false discovery rate) choice of 5% and are derived from *coding region prediction only* (i.e. from *CS-coding*). We excluded samples with evidence of hypermutation in the determination of the results in Figures 1 to 5 (main paper). Our criterion for exclusion was prediction of more than 500 SNV-drivers in *coding regions*. We did not include predictions from non-coding regions in our estimation of a prospective hypermutation example because of the weak performance of the non-coding predictor (*CS-noncoding*) [1] and the poor extent of known functionality of non-coding genomic regions. Skin cutaneous melanoma (SKM) had the highest proportion of hypermutators at 36.7%. Next were gastric adenocarcinoma (STAD) and colon adenocarcinoma (COAD) at 22.6% and 20.9% respectively. In the Table we state zero-count instances for prediction in coding regions and zero-count instances were *included* in counts. For the majority of cancer types there are only a limited number of instances with a SNV-driver count of zero, in coding regions. Thyroid cancer, with its very low overall mean count, could be expected to have a higher number of these but the proportion is only 6.6% (for a FDR of 5%). Neuroblastoma, though, also has a similarly low mean count for SNV-drivers and the proportion of samples with a zero count is high at 46.0%. This may indicate a more crucial role for other types of drivers with this disease, beyond single point mutations.

Cancer subtype	Typecode	Sample size	Hypermutators	Zero-count
Bladder Urothelial	BLCA	233	12	0
Breast	BRCA	150	3	2
Cervical Squamous Cell Carcinoma	CESC	194	12	5
Colon Adenocarcinoma	COAD	215	45	0
Colorectal	COCA	187	18	6
Early Onset Prostate	EOPC	62	0	22
Esophageal	ESCA	228	0	15
Gastric	GACA	9	0	0
Kidney Renal Clear Cell Carcinoma	KIRC	404	0	2
Kidney Renal Papillary Cell Carcinoma	KIRP	159	0	0
Brain Lower Grade Glioma	LGG	283	1	6
Liver	LICA	421	3	20
Liver Hepatocellular Carcinoma	LIHC	188	2	1
Malignant Lymphoma	MALY	100	1	1
Neuroblastoma	NBL	106	0	46
Oral	ORCA	131	2	1
Ovarian	OV	181	0	0
Pancreatic	PACA	687	16	10
Prostate	PRAD	488	2	32
Rectum Adenocarcinoma	READ	79	4	0
Renal Cell	RECA	105	0	6
Skin Cutaneous Melanoma	SKCM	335	123	2
Gastric Adenocarcinoma	STAD	288	65	0
Thyroid	THCA	528	1	35
Uterine Corpus Endometrial Carcinoma	UCEC	246	36	1

Table 1: This Table gives the numbers of samples (drawn from the International Cancer Genome Consortium dataset [5]) used as test data in our study (under sample size), followed by the number of hypermutators and numbers with zero counts for SNV-drivers (for a FDR of 5%) in the latter two columns (for coding region prediction). Samples exhibiting potential hypermutation were excluded from our study, instances where zero SNV-driver counts were predicted, were included.

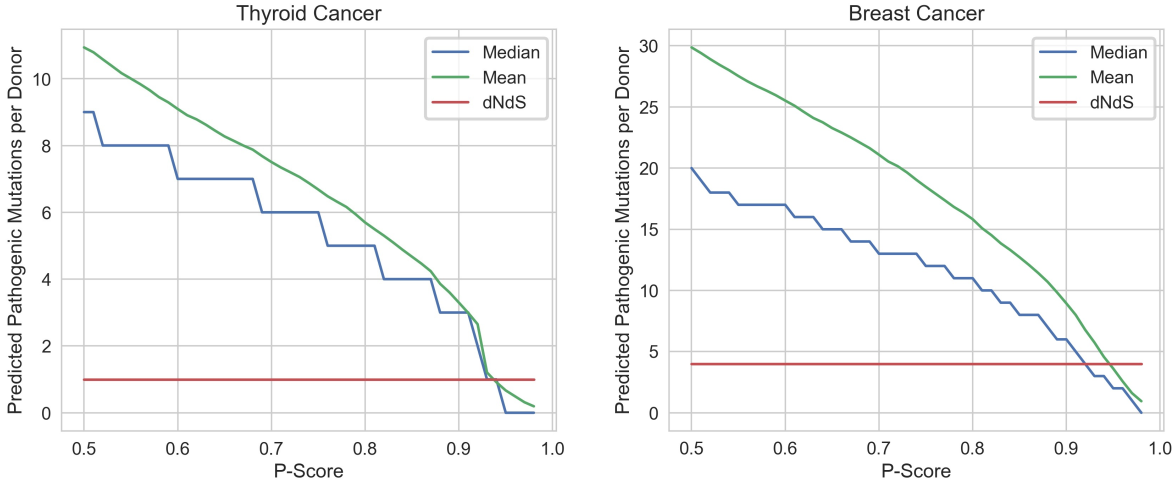

Figure 1: The median, mean and dN/dS predictions for **right**: breast cancer and **left**: thyroid cancer. The mean or median number of number of disease-driver mutations (y-axis) is plotted against the threshold on the p-score (x-axis) which is an estimation of the confidence in a class assignment to positive (disease-driver) status for a variant identified in the cancer genome. The horizontal line is the estimate of the mean proposed by Martincorena *et al* [7]. If we lower the threshold on the confidence (p-score) we allow through more positive predictions.

2 Threshold dependency of the counts and comparison with other methods

In Supplementary Figure 1 we plot the median and means for the predicted numbers of SNV-drivers in coding regions of the breast cancer (left) and thyroid cancer (right) genome. The horizontal line is the estimate from Martincorena *et al* [7]. At a threshold on the p-score of 0.9 the estimates are in approximate agreement. However, as noted in the main text, if we should make a less stringent choice on the p-score (the confidence in the prediction) then this lets through more positive (disease-driver) predictions. However, retaining this choice for the p-score threshold (0.9), we see from Supplementary Figure 2, that there is an approximate agreement on the cancer types with the smallest mean for the SNV-driver counts (e.g. thyroid cancer) and the largest (e.g. bladder urothelial cancer). *CScape* consistently gives higher mean and median counts over Martincorena *et al* [7]. However, the concept that the sizes of coding SNV-driver sets is relatively small is confirmed.

3 Additional plots complementing Figure 3 of the main paper

We give two additional plots below complementing Figure 3 of the main text. In Supplementary Figure 3 we present the full set of curves for the mean counts. In Supplementary Figure 4 we present the full set of curves of the median counts of SNV-drivers across all cancers (since only a selection is presented in Figure 3 of the main paper).

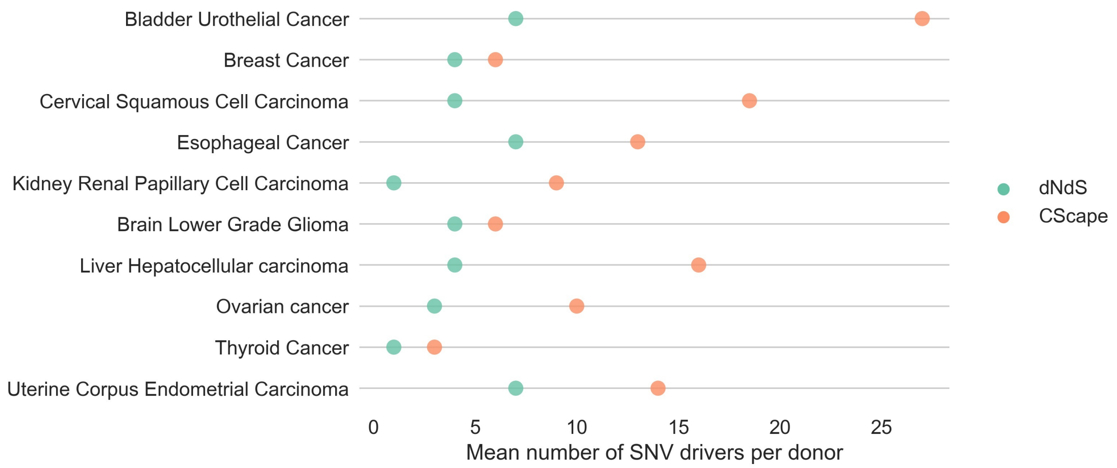

Figure 2: The mean number of SNV-drivers from Martincorena *et al* [7] (green) and the mean number of SNV-drivers from *CScape* (orange) for a variety of common solid tumours, for single nucleotide variants in coding regions of the cancer genome. These results are presented for a threshold of 0.9 on the p-scores from *CScape* and include those cancer types covered by Martincorea *et al* [7].

4 Estimating the number of SNV-drivers by stage of disease

Data was extracted from the International Cancer Genome Consortium database [5]. Only in a subset of instances were we able to extract the clinical annotation by stage and the data used for constructing Supplementary Tables 2 and 3 therefore differs from, and is a subset of, data used for deriving the figures in the main paper. Not all cancer samples have been staged since some cancer staging requires molecular characteristics to be taken into account (for example, breast cancer): these cancers were omitted from the staging analysis. In line with our discussion in the main paper we used a cutoff on the p-score of 0.88 in coding regions.

A trend towards increasing numbers of SNV-drivers with increasing stage of disease is not well established for malignant lymphoma, oral, pancreatic cancer, neuroblastoma, renal and thyroid cancer. For neuroblastoma, thyroid and renal cancer the numbers of SNV-drivers is low with initial stage of disease and remains fairly constant and low throughout. For other cancers there is a more pronounced trend towards increasing numbers of SNV-drivers with stage of disease. This observation could be applied to colorectal cancer where the numbers of SNV-drivers evolves from a mean of 16.6 for Stage I to 42.6 at Stage IV, supported by large sample sizes at each stage. Liver cancer is another cancer with increasing number of SNV-drivers with stage of disease. Finally, both early onset (EOPC) and late onset prostate cancer (PRAD) have a systematic trend of increasing numbers of SNV-drivers as we proceed from early stage to late stage disease.

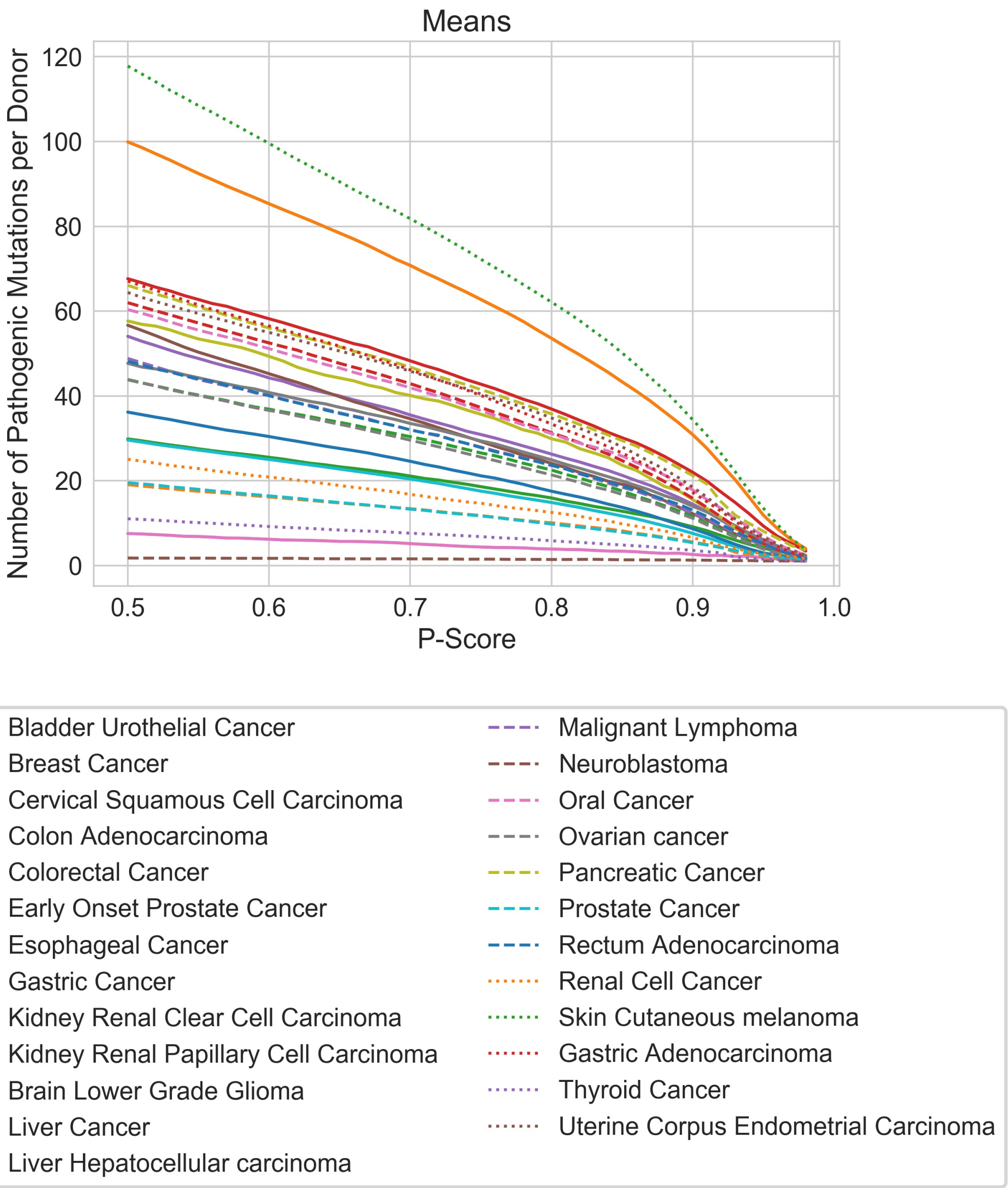

Figure 3: A plot of the means (y-axis) versus the p-score threshold (x-axis) across the full range of common solid tumours. This Figure complements Figure 3 of the main paper which gives the median counts for SNV-drivers across the same range of common solid tumours. Relative to the plot with the median counts (main paper, Figure 3) there is some shift in the relative ordering of different type of cancer in terms of driver counts.

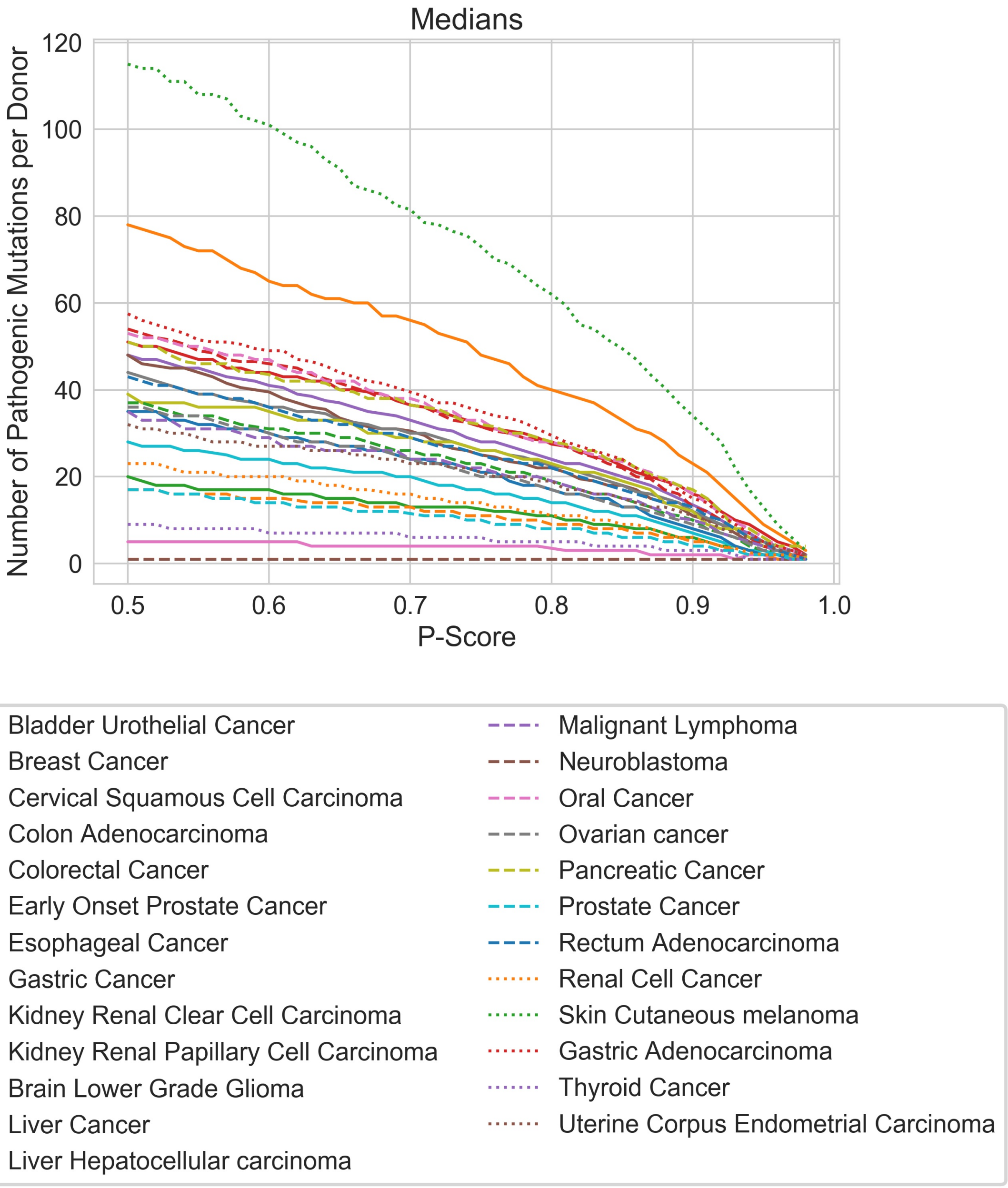

Figure 4: A plot of the medians (y-axis) versus the p-score threshold (x-axis) for all the cancers considered in the main paper. This Figure complements Figure 3 of the main paper which gives the median counts for SNV-drivers across a selection of common solid tumours.

Name or Stage	Mean	Lower Quartile	Upper Quartile	Sample Size
**1. Bladder Urothelial (BLCA)**				
0a	11.5	7.3	15.7	6
I	12.9	9.3	16.4	31
II	15.2	9.6	20.8	29
III	12.9	7.7	18.1	14
IIIA	15.7	10.1	21.4	21
**2. Colorectal (COCA)**				
I	16.6	8.3	24.9	40
IIA	27.8	15.5	40.2	49
IIB	25.4	15.0	35.8	46
IIIA	7.0	4.4	9.6	8
IIIB	19.4	12.2	26.6	77
IIIC	31.5	8.4	54.6	27
IVA	42.6	18.6	66.6	49
**3. Early Onset Prostate Cancer (EOPC)**				
I	2.5	1.9	3.1	102
IIA	3.8	1.5	6.2	42
IIIB	6.5	4.4	8.6	2
**4. Esophageal (ESCA)**				
I	13.6	7.5	19.6	12
II	9.9	8.9	10.9	135
III	10.0	8.5	11.6	92
IVA	12.2	9.1	15.3	16
**5. Gastric (GACA)**				
IA	16.6	1.3	31.9	8
IB	3.8	3.4	4.1	5
II	10.0	10.0	10.0	1
IV	10.2	6.0	14.4	65
**6. Liver (LICA)**				
IA	45.6	24.5	66.7	31
II	66.9	6.9	127.0	13
IIIA	169.2	33.3	305.1	5
IVB	135.0	135.0	135.0	1
**7. Malignant Lymphoma (MALY)**				
I	7.4	5.2	9.6	30
II	11.4	6.5	16.3	34
III	9.2	6.9	11.4	79
IV	6.8	5.6	8.0	70

Table 2: A list a seven cancer types with the frequency counts for the drivers stratified by stage.

Name or Stage	Mean	Lower Quartile	Upper Quartile	Sample Size
**8. Neuroblastoma (NBL)**				
IIA	1.0	1.0	1.0	1
III	1.3	0.8	1.9	3
IV	1.1	1.0	1.2	3
**9. Oral (ORCA)**				
II	13.3	7.8	18.7	4
III	14.8	4.3	25.2	8
IVA	11.1	9.5	12.7	112
IVB	24.0	24.0	24.0	1
**10. Ovarian (OV)**				
III	9.4	7.9	11.0	78
IV	10.3	5.0	15.6	14
**11. Pancreatic (PACA)**				
IA	16.9	12.9	21.0	12
IB	20.3	14.4	26.3	44
II	12.0	7.8	16.2	4
IIA	29.1	5.5	52.7	30
IIB	24.8	15.7	34.0	74
III	21.7	14.7	28.6	9
IV	17.0	8.1	25.9	3
**12. Prostate (PRAD)**				
IIB	4.4	3.9	4.9	301
IIC	7.6	4.7	10.5	13
IIIB	16.4	9.1	23.7	19
IVA	21.8	6.8	36.6	4
**13. Renal Cell (RECA)**				
I	3.4	2.7	4.1	114
II	3.3	2.2	4.5	33
III	3.2	2.2	4.2	56
IV	3.0	2.1	4.0	34
**14. Thyroid (THCA)**				
I	2.0	1.7	2.4	55
II	2.4	1.2	3.7	7
III	2.1	1.7	2.6	24

Table 3: A second list a seven cancer types with the frequency counts for the drivers stratified by stage.

5 The top ranked driver genes according to cancer type

In this section we present the top five driver genes categorised according to cancer type, for the 25 cancer types listed in Table 1 of this Supplementary. To identify these genes we have used the *CScape* classifier on data from the International Cancer Genome Consortium [5]. Since *CScape* was trained on COSMIC [2] and 1000 Genomes [3] data, this constitutes an independent test set. A variant was labelled as a driver if the associated p-score for the confidence in that status exceeded 0.88. This value for the p-score cutoff was selected because it gives a false discovery rate (FDR) of 5% (see main paper, Section 2). If a gene had at least one such SNV-driver, we incremented the sum and divided the final total by the number of donor samples considered for that cancer type. Given that sample sizes (number of donors) are generally quite large, the differences between occurrence rates of such driver mutations by gene are very statistically significant.
The lists below cover the top five genes by type of cancer, as discussed in the main paper. At the *CScape* website (http://cscape.biocompute.org.uk/), under the Help/Documentation webpage, we give a downloadable file (*driver-genes*) which gives these ranked genes down to the level of no SNV-drivers in the gene with confidence greater than 0.88.

**1.**	**Bladder Urothetial**	**BLCA**
Gene name	No. with a $p \geq 0.88$/Total no. donors	Percentage
$MUC4$	58/236	24.5
TTN	31/236	13.1
$TTN-AS1$	30/236	12.7
$TP53$	28/236	11.9
$PIK3CA$	24/236	10.2
**2.**	**Breast**	**BRCA**
Gene name	No.with a $p \geq 0.88$/Total no. donors	Percentage
$TP53$	42/191	22.0
$PIK3CA$	17/191	8.9
TTN	11/191	5.8
$TTN-AS1$	10/191	5.2
$AKT1$	9/191	4.7
**3.**	**Cervical Squamous Cell Carcinoma**	**CESC**
Gene name	No.with a $p \geq 0.88$/Total no. donors	Percentage
$PIK3CA$	47/194	24.2
TTN	26/194	13.4
$TTN-AS1$	26/194	13.4
$MUC4$	26/194	13.4
$KMT2C$	22/194	11.3
**4.**	**Colon Adenocarcinoma**	**COAD**
Gene name	No. with a $p \geq 0.88$/Total no. donors	Percentage
APC	124/253	49.0
$KRAS$	103/253	40.7
$CTC-554D6.1$	101/253	39.9
$TP53$	82/253	32.4
TTN	58/253	22.9
**5.**	**Colorectal**	**COCA**
Gene name	No. with a $p \geq 0.88$/Total no. donors	Percentage
APC	125/319	39.2
$KRAS$	106/319	33.2
$CTC-554D6.1$	104/319	32.6
$TP53$	97/319	30.4
TTN	49/319	15.3
**6.**	**Early onset prostate**	**EOPC**
Gene name	No. with a $p \geq 0.88$/Total no. donors	Percentage
$TP53$	10/202	5.0
$RYR2$	6/202	3.0
$REM1$	4/202	2.0
$LRP1B$	4/202	2.0
$LOXHD1$	4/202	2.0

Table 4: Top 5 ranked driver genes for bladder urothelial, breast, cervical, colon adenocarcinoma, colorectal and early onset prostate cancer.

**7.**	**Esophageal**	**ESCA**
Gene name	No. with a $p \geq 0.88$/Total no. donors	Percentage
$TP53$	163/330	49.3
TTN	26/330	7.8
$PIK3CA$	24/330	7.3
$TTN-AS1$	23/330	7.0
$CSMD3$	22/330	6.7
**8.**	**Gastric**	**GACA**
Gene name	No. with a $p \geq 0.88$/Total no. donors	Percentage
$TP53$	23/92	25.0
$LRP1B$	8/92	8.7
$KMT2C$	6/92	6.6
$CSMD3$	5/92	5.4
$UNC80$	4/92	4.3
**9.**	**Kidney Renal Clear Cell Carcinoma**	**KIRC**
Gene name	No. with a $p \geq 0.88$/Total no. donors	Percentage
$MUC4$	57/408	14.0
$PBRM1$	46/408	11.3
VHL	41/408	10.0
TTN	19/408	4.7
$TTN-AS1$	17/408	4.2
**10.**	**Kidney Renal Papillary Cell Carcinoma**	**KIRP**
Gene name	No. with a $p \geq 0.88$/Total no. donors	Percentage
$MUC4$	11/165	6.7
TTN	9/165	5.5
$TTN-AS1$	8/165	4.8
MET	7/165	4.2
$SMARCA4$	5/165	3.0
**11.**	**Brain Lower Grade Glioma**	**LGG**
Gene name	No. with a $p \geq 0.88$/Total no. donors	Percentage
$IDH1$	215/283	76.0
$TP53$	79/283	25.4
$PIK3CA$	17/283	6.0
$RP11-799N11.1$	15/283	5.3
$CTC-297N7.11$	15/283	5.3
**12.**	**Liver**	**LICA**
Gene name	No. with a $p \geq 0.88$/Total no. donors	Percentage
$TP53$	66/399	16.5
TTN	59/399	14.8
$TTN-AS1$	56/399	14.0
$LRP1B$	48/399	12.0
$RYR2$	47/399	11.8

Table 5: Top 5 ranked driver genes for esophageal, gastric, kidney renal clear cell carcinoma, brain lower grade glioma and liver cancer.

**13.**	**Liver Hepatocellular Carcinoma**	**LIHC**
Gene name	No. with a $p \geq 0.88$/Total no. donors	Percentage
$CTNNB1$	43/189	22.8
$TP53$	30/189	15.9
TTN	19/189	10.1
$TTN-AS1$	18/189	9.6
$UNC80$	12/189	6.3
**14.**	**Malignant Lymphoma**	**MALY**
Gene name	No. with a $p \geq 0.88$/Total no. donors	Percentage
$CREBBP$	54/241	22.4
$KMT2D$	48/241	19.9
$EZH2$	36/241	14.9
$TP53$	32/241	13.3
$STAT6$	21/241	8.7
**15.**	**Neuroblastoma**	**NBL**
Gene name	No. with a $p \geq 0.88$/Total no. donors	Percentage
ALK	10/205	4.9
$PTPN11$	5/205	2.4
$RP11-799N11.1$	4/205	2.0
$NF1$	4/205	2.0
$CTC-297N7.11$	4/205	2.0
**16.**	**Oral**	**ORCA**
Gene name	No. with a $p \geq 0.88$/Total no. donors	Percentage
$TP53$	55/125	44.0
$FAT1$	25/125	20.0
$NOTCH1$	19/125	15.2
TTN	17/125	13.6
$TTN-AS1$	17/125	13.6
**17.**	**Ovarian**	**OV**
Gene name	No. with a $p \geq 0.88$/Total no. donors	Percentage
$TP53$	114/211	54.0
TTN	13/211	6.2
$TTN-AS1$	12/211	5.7
$CSMD3$	12/211	5.7
$CTC-297N7.11$	9/211	4.2
**18.**	**Pancreatic**	**PACA**
Gene name	No. with a $p \geq 0.88$/Total no. donors	Percentage
$KRAS$	563/651	86.5
$TP53$	266/651	41.0
$SMAD4$	84/651	12.9
TTN	41/651	6.3
$TTN-AS1$	38/651	5.8

Table 6: Top 5 ranked driver genes for liver hepatocellular carcinoma, malignant lymphoma, neuroblastoma, oral, ovarian and pancreatic cancer.

**19.**	**Prostate**	**PRAD**
Gene name	No. with a $p \geq 0.88$/Total no. donors	Percentage
$SPOP$	42/684	6.1
$TP53$	31/684	4.5
$MUC4$	17/684	2.5
TTN	15/684	2.1
$TTN-AS1$	14/684	2.0
**20.**	**Rectum Adenocarcinoma**	**READ**
Gene name	No. with a $p \geq 0.88$/Total no. donors	Percentage
APC	58/93	62.4
$CTC-554D6.1$	53/93	57.0
$TP53$	41/93	44.1
$KRAS$	38/93	41.0
TTN	19/93	20.4
**21.**	**Renal cell**	**RECA**
Gene name	No. with a $p \geq 0.88$/Total no. donors	Percentage
$PBRM1$	56/388	14.4
VHL	50/388	12.9
$SETD2$	27/388	7.0
$MTOR$	26/388	6.7
$BAP1$	17/388	4.4
**22.**	**Skin Cutaneous Melanoma**	**SKCM**
Gene name	No. with a $p \geq 0.88$/Total no. donors	Percentage
TTN	172/335	51.3
$TTN-AS1$	164/355	46.1
$CTC-297N7.11$	150/355	42.3
$BRAF$	150/355	42.3
$RP11-799N11.1$	146/355	41.1
**23.**	**Gastric Adenocarcinoma**	**STAD**
Gene name	No. with a $p \geq 0.88$/Total no. donors	Percentage
TTN	78/287	27.2
$TTN-AS1$	73/287	25.4
$TP53$	73/287	25.4
$CSMD3$	44/287	15.3
$LRP1B$	39/287	13.6
**24.**	**Thyroid**	**THCA**
Gene name	No. with a $p \geq 0.88$/Total no. donors	Percentage
$BRAF$	303/543	55.8
TTN	7/543	1.3
$TTN-AS1$	7/543	1.3
$NRAS$	7/543	1.3
$KMT2C$	7/543	1.3

Table 7: Top 5 ranked driver genes for prostate, rectum adenocarcinoma, renal, skin cutaneous melanoma, gastric adeoncarcinoma and thyroid cancer.

**25.**	**Uterine Corpus Endometrial Carcinoma**	**UCEC**
Gene name	No. with a $p \geq 0.88$/Total no. donors	Percentage
$PIK3CA$	88/250	35.2
$PTEN$	80/250	32.0
$CTNNB1$	69/250	27.6
$ARID1A$	54/250	21.6
$KRAS$	50/250	20.0

Table 8: Top 5 ranked driver genes for uterine corpus endometrial carcinoma.

6 Prediction on non-coding disease-drivers

We pursued a study of non-coding SNV-drivers proposed in the Pan-Cancer Analysis of Whole Genomes (PCAWG) study of Rheinbay *et al* [10]. The dataset used is derived from the International Cancer Genome Consortium [5] and the The Cancer Genome Atlas [6] and independent of the datasets used to train CScape (COSMIC [2] and 1000 Genomes [3]). The results are tabulated in Supplementary Table 9 and derive from listed prospective non-coding drivers available among their list of the top 50 single point mutations drivers (Extended Data Figure 1 in [10]). A restriction has been made to prospective drivers located on autosomes and labelled as residing in non-coding regions of the cancer genome.

Chr.	Position	Ref.	Mut.	Confidences	Prediction
5	1295228	G	{A,C,T}	{0.491,0.637,0.727}	Possibly oncogenic
12	25389285	T	{A,C,G}	{0.717,0.477,0.578}	Possibly oncogenic
5	1295250	G	{A,C,T}	{0.543,0.557,0.669}	Oncogenic
14	106326944	C	{A,G,T}	{0.682,0.662,0.558}	Oncogenic
19	49990694	G	{A,C,T}	{0.690,0.536,0.708}	Oncogenic
14	106326944	C	{A,G,T}	{0.682,0.662,0.558}	Oncogenic
19	49990694	G	{A,C,T}	{0.690,0.536,0.708}	Oncogenic
14	106326944	C	{A,G,T}	{0.682,0.662,0.558}	Oncogenic
14	106326713	G	{A,C,T}	{0.636,0.632,0.681}	Oncogenic
14	106328942	G	{A,C,T}	{0.465,0.567,0.647}	Possibly oncogenic
6	142706206	G	{A,C,T}	{0.781,0.814,0.827}	Oncogenic
8	56987141	C	{A,G,T}	{0.730,0.605,0.608}	Oncogenic
14	106329192	G	{A,C,T}	{0.622,0.672,0.725}	Oncogenic
14	106326887	C	{A,G,T}	{0.575,0.587,0.600}	Oncogenic
14	106326619	C	{A,G,T}	{0.670,0.652,0.589}	Oncogenic
14	106326618	G	{A,C,T}	{0.622,0.632,0.681}	Oncogenic
14	106327115	G	{A,C,T}	{0.265,0.505,0.702}	Possibly oncogenic
14	106327559	G	{A,C,T}	{0.664,0.483,0.388}	Possibly oncogenic
14	106240243	G	{A,C,T}	{0.633,0.599,0.661}	Oncogenic
3	164903710	T	{A,C,G}	{0.711,0.531,0.709}	Oncogenic
14	106329196	C	{A,G,T}	{0.706,0.547,0.683}	Oncogenic
14	106329852	C	{A,G,T}	{0.683,0.664,0.554}	Oncogenic
14	106329550	G	{A,C,T}	{0.530,0.674,0.720}	Oncogenic
10	115511590	G	{A,C,T}	{0.702,0.740,0.734}	Oncogenic
10	115511593	C	{A,G,T}	{0.565,0.648,0.684}	Oncogenic
19	2151793	C	{A,G,T}	{0.719,0.721,0.696}	Oncogenic
14	106326877	T	{A,C,G}	{0.518,0.401,0.398}	Possibly oncogenic
14	106329236	G	{A,C,T}	{0.486,0.517,0.616}	Possibly oncogenic
14	106329350	C	{A,G,T}	{0.674,0.657,0.596}	Oncogenic
14	106327417	C	{A,G,T}	{0.527,0.560,0.670}	Oncogenic
1	103599442	T	{A,C,G}	{0.436,0.306,0.294}	Benign

Table 9: The top commonly recurrent single point driver mutations in *non-coding* regions proposed by Rheinbay *et al* (Extended Data Figure 1 in [10]). This table only gives single nucleotide variants located on autosomes and labelled by our classifier as residing in non-coding regions. The table presents the chromosome (Chr.), position and reference nucleotide (Ref.) based on the GRCh37 reference genome. The three prospective variants are presented (Mut.) with the confidence of driver-status given in the next column, in the same relative order, and derived from our predictor *CScape* (*http://cscape.biocompute.org.uk*)). Mutation at a position is labelled *oncogenic* if all three variants from reference are predicted as having disease-driver status. Mutation at a position is labelled *possibly oncogenic* if some variants from reference are predicted as having disease-driver status.

References

[1] Rogers, M., Shihab, H. A., Gaunt, T. R. & Campbell, C. CScape: a tool for predicting oncogenic single-point mutations in the cancer genome. *Scientific Reports (Nature)* **7**, 11597 (2017).

[2] http://cancer.sanger.ac.uk/cosmic/help/gene/analysis.

[3] The 1000 Genomes Project Consortium. An integrated map of genetic variation from 1,092 human genomes. *Nature* **491**, 56–65 (2012).

[4] Campbell, C. & Ying, Y. *Learning with Support Vector Machines* (Morgan and Claypool, 2011).

[5] https://icgc.org/icgc.

[6] https://tcga-data.nci.nih.gov.

[7] Martincorena, I. *et al.* Universal patterns of selection in cancer and somatic tissues. *Cell* **171**, 1029–1041 (2017).

[8] Schapire, R. E The boosting approach to machine learning: An overview. *Nonlinear estimation and classification* 149–171,Springer(2003).

[9] Hindroff, L. A. *et al.* Potential etiologic and functional implications of genome-wide association loci for human diseases and traits. *Proc. Natl. Acad. Sci. U.S.A.* **106**, 9362–9367 (2009).

[10] Rheinbay, E. *et al.* Discovery and characterization of coding and non-coding driver mutations in more than 2,500 whole cancer genomes. *bioRxiv* (2017).
